# Estimates of Total Dietary Folic Acid Intake in the Australian Population Following Mandatory Folic Acid Fortification of Bread

**DOI:** 10.1155/2012/492353

**Published:** 2012-08-23

**Authors:** Jacinta Dugbaza, Judy Cunningham

**Affiliations:** Food Composition Evaluation and Modelling Section, Food Standards Australia New Zealand (FSANZ), 55 Blackall Street, Barton Canberra, ACT 2600, Australia

## Abstract

Mandatory folic acid fortification of wheat flour for making bread was implemented in Australia in September 2009, to improve the dietary folate status of women of child-bearing age, and help reduce the incidence of neural tube defects in the population. This paper presents estimates of folic acid intake in the target population and other subgroups of the Australian population following implementation of the mandatory folic acid fortification standard. In June/July 2010 one hundred samples from seven bread categories were purchased from around the country and individually analysed for the amount of folic acid they contained. A modification to the triple enzyme microbiological method was used to measure folic acid in the individual bread samples. The folic acid analytical values together with national food consumption data were used to generate estimates of the population's folic acid intake from fortified foods. Food Standards Australia New Zealand's (FSANZ) custom-built dietary modelling program (DIAMOND) was used for the estimates. The mean amount of folic acid found in white bread was 200 **μ**g/100 g which demonstrated that folic-acid-fortified wheat flour was used to bake the bread. The intake estimates indicated an increase in mean folic acid intake of 159 **μ**g per day for the target group. Other sub-groups of the population also showed increases in estimated mean daily intake of folic acid.

## 1. Introduction

This paper focuses on estimates of the Australian population's (including women of child bearing age, the target group for folic acid fortification) intake of folic acid from voluntary and mandatory fortified foods, and not their intake of total dietary folate from all foods consumed. Folic acid as used in this paper refers to added folic acid in the fortified foods reported as consumed by the respondents of the two national food consumption datasets used for the dietary modelling. Folic acid is the chemical form of folate normally used by the food industry as a fortificant.

FSANZ is able to estimate the target population's intake of folic acid because the *Australia New Zealand Food Standards Code *(the Code) clearly specifies foods that can be fortified under the voluntary and mandatory fortification permissions. Before implementation of the mandatory folic acid fortification standard, specific foods in the Australian market could be fortified under the voluntary folic acid permission outlined in Standard 1.3.2 of the Code. It was therefore possible to identify these foods that individuals reported as consumed and apply a factor accounting for the relevant market share of fortified to unfortified product in the dietary modelling, using data obtained from the food industry for each food category. Where permissions for foods or food groups to be voluntarily fortified with folic acid had not been taken up by industry, these foods or food groups were not included in the model. In addition, because the mandatory folic acid fortification standard relates only to wheat flour for baking bread, it was possible to identify foods consumed that have been fortified with folic acid under the mandatory standard. The estimates did not take into account naturally occurring folates in the foods consumed by the respondents, or folic acid from the use of folic acid supplements or multivitamin supplements containing folic acid. 

In developing the folic acid fortification standard, estimates of natural folate and total dietary folate equivalent intakes for the Australian population were previously presented by FSANZ and are not further discussed here [1]. Although the total folate contents of the bread samples were also determined, they are not presented in this paper but were used to validate our folic acid content results and for updating the national food composition database, which FSANZ maintains [2]. 

Australia implemented mandatory folic acid fortification of wheat flour for making bread in September 2009. The purpose was to reduce the incidence of neural tube defects (NTDs) by increasing the intake of dietary folic acid among women of child-bearing age (16–44 years), the target population.

It had been estimated that between 300 and 350 pregnancies were affected by NTDs each year in Australia [3] and that the numbers were higher in Indigenous communities [4]. The total dietary folate intake of the target population had remained well below recommended levels despite the various health programs that had been initiated since 1993 to encourage women of child-bearing age to increase their intake of foods high in natural folates and/or folic acid supplements [5]. 

In Australia, the mandatory folic acid fortification standard applies only to wheat flour for making bread. However, the standard exempts wheat flour for making bread represented as “organic” from the fortification requirement. The exemption allows the organic wheat flour milling industry and bread manufacturers to comply with the fair trading legislation (Australia, *Trades Practices Act 1974*), which takes precedence over the Code. 

Wheat flour for making bread was selected as the food vehicle for fortification because bread is widely consumed by the population, including the target group. In proposing the mandatory folic acid fortification level for wheat flour, FSANZ considered a number of national scenarios, available scientific evidence, and the experiences of other developed countries that had instituted mandatory folic acid fortification of flour. The fortification level was set as a range of 200–300 *μ*g folic acid per 100 g wheat flour. The Australian level has been set as a range to reduce industry overages, taking into account that a degree of uncertainty exists with regard to the effects of long-term increased folic acid intake by the nontarget population, especially children.

Countries such as the United States of America (USA) and Canada that have mandated folic acid fortification of wheat flour have not prescribed a fortification range. For example, the USA food standard requires each pound of enriched flour (fortified flour) to contain 0.7 milligrams of folic acid [6]. This translates to approximately 154 *μ*g folic acid per 100 g of flour. In Canada flour, white flour, enriched flour, or enriched white flour are required to contain 150 *μ*g folic acid per 100 g of flour [7].

In Australia, the mandated level of folic acid in wheat flour for making bread was expected to increase the average intake of folic acid among the target group by 100 *μ*g/day. This would be above the levels already achieved through use of foods voluntarily fortified with folic acid and use of dietary supplements. It had been estimated that the target population's intake of folic acid from voluntarily fortified foods (including breakfast cereals and yeast-based spreads) was about 108 *μ*g folic acid per day. The estimated additional 100 *μ*g folic acid from mandatory fortification of bread was expected to increase total folic acid intake (from voluntary and mandatory fortified foods) and bring about the reduction in number of NTD-affected pregnancies by up to 14%. 

FSANZ's role in determining the postfortification levels of folic acid in bread and assessing the population's (including the target group) intake of dietary folic acid is part of the national activities to monitor the mandatory folic acid fortification standard, which includes monitoring of NTD affected pregnancies. The monitoring activities for Australia and New Zealand are described in two recent reports published by the Australian Institute of Health and Welfare (AIHW) [8], with pre-fortification data for the prevalence of NTDs in Australia also recently published by the AIHW [9].

## 2. Materials and Methods

### 2.1. Folic Acid in Bread

To measure the amounts of folic acid in breads sold in the country, a survey of breads was undertaken in June/July 2010, nine to ten months after implementation of the mandatory fortification standard. One hundred samples from seven bread categories were purchased from grocery shops and bakeries in the capital cities of all states and territories. The bread types were selected based on both their market share and on the need to capture the types generally consumed nationwide.

Samples were transported under refrigeration to the National Measurement Institute's Analytical Laboratories in Melbourne, Australia. At the laboratory, each individual loaf was weighed (all slices plus the two crusts) and divided into two halves. One half was left to dry at ambient temperature and the other labelled and frozen. Following the drying, all the samples were individually reweighed, homogenised thoroughly and stored in labelled air-tight containers to be used for the required chemical analyses. A portion of the homogenised material for each bread sample was then taken and prepared for folic acid and total folate analyses. No composite samples were used. 

The amount of folic acid in the samples was determined using a modified form of the triple enzyme microbiological method AOAC 2004.5 [10] that is accredited by the National Accreditation Association of Testing Authorities, Australia (NATA). The method eliminated the protease and conjugase digestion steps from the tri-enzyme digestion for measuring total folate using *Lactobacillus casei* (spp *rhamnosus*) ATCC 7469 [11]. The limit of detection for folic acid was 3 *μ*g/100 g. The results obtained provided information on the amount of folic acid present in each of the 100 individual bread samples purchased. The folic acid analytical results from the bread survey were used for the intake assessment.

### 2.2. Folic Acid in Other Foods

To determine folic acid intake before mandatory fortification, the nutrient data released in AUSNUT 2007 [12] were used to estimate folic acid intakes from voluntarily fortified foods such as breakfast cereals, juices, and yeast-based spreads. Most of the folic acid values in AUSNUT for fortified foods were determined using the same triple enzyme microbiological method, although some were developed using imputation and recipe calculations.

### 2.3. Food Consumption Data Used

Food consumption data from 24-hour recalls of the 1995 Australian National Nutrition Survey (1995 NNS) [13] and the 2007 Australian National Children's Nutrition and Physical Activity Survey (2007 ANCNPAS) [14] were used in estimating the dietary intake of folic acid by the target group and other sub-groups of the population.

The 1995 NNS sampled approximately 13,858 respondents aged two years and above from urban and rural areas in all states/territories, from February 1995 to March 1996. Only residents of private dwellings were sampled. Approximately 10% of the NNS participants provided a second 24-hour period food recall on a nonconsecutive day.

The 2007 ANCNPAS collected data on food and nutrient intake, physical activity levels, and physical measurements from 4,487 children aged 2–16 years across Australia. Participants were asked to recall all food, drink, and dietary supplements they had consumed in the previous 24 hours. In a follow-up telephone interview held one to three weeks later, participants were again asked to recall food, drink, and supplements consumed the previous day. The survey used stratified sampling with nonproportional samples, and was conducted between February and August 2007.

In both surveys, each food and beverage consumed was described in sufficient detail to allow its nutrient composition to be determined. For this investigation, only data for those aged 17 years and above was used from the 1995 NNS because of the newer food consumption data from the 2007 ANCNPAS for 2 to 16 year olds.

### 2.4. Dietary Modelling Methodology

FSANZ's custom-built dietary modelling program (DIAMOND) was used to estimate folic acid intakes. The average of two days' food consumption data was used to better estimate “usual” intake for population groups before and after mandatory fortification. It was not considered appropriate to use a statistical adjustment method to estimate “usual” intake due to the nonnormality of the distribution of folic acid intakes [15].

Because the 1995 NNS data captured two days of food consumption for only 10% of respondents, the data used to estimate the two-day average folic acid intakes were for only those respondents with both day 1 and day 2 food recalls. This restricted the number of respondents available for analysis, and the estimates produced may not be fully representative of the total population of women of child-bearing age. For example, while there were 3,178 female respondents aged 16–44 years in the 1995 NNS data, the number of consumers in the age group was reduced to 328 when food consumption data for only those with both day 1 and day 2 recalls were compiled. Similarly, although there were 10,851 consumers in the 19+ age group for day 1 consumption data, the number was reduced to 1,163 when data were compiled for those with both day 1 and day 2 food recalls. 

The estimated dietary folic acid intakes were based on the amounts of folic acid in the fortified foods consumed by the respondents in the 1995 and 2007 surveys. Data were available for the folic acid content of breads that had been mandatorily fortified, foods that had been voluntarily fortified, and mixed foods containing bread or other folic-acid-fortified foods as ingredients. For each individual included in the dietary modelling, daily intake of folic acid (*μ*g/day) was estimated by multiplying the concentration of folic acid (*μ*g/100 g) in each fortified food by the amount of that food consumed (g/day), and summing this across all the fortified foods consumed.

Intake estimates were generated for two scenarios, before (voluntary fortification only) and after mandatory fortification of wheat flour for making bread. The estimates do not include the contribution of dietary supplements to intake. Data were not available for the 1995 NNS and although data on supplement consumption were available from the ANCNPAS, few children consumed supplements containing folic acid.

## 3. Results

### 3.1. Postfortification Amounts of Folic Acid in Bread

The mean amount of folic acid in the five main bread categories (white sandwich bread, wholemeal breads, multigrain and seeds bread, English muffins and flat breads/wraps) was 185 *μ*g per 100 g bread (range 165 to 200 *μ*g/100 g). The lowest levels were found in gluten-free and organic breads which are not required to be made from folic-acid-fortified wheat flour (see Table 1).

Factors that are likely to have influenced the measured levels of added folic acid include:

- the proportion of folic-acid-fortified flour used in the bread recipe, which is higher in white breads than in grain and seed breads, and in flat breads compared to sandwich loaf breads;
- flour production practices in Australia, where wholemeal flour is produced from fortified white flour to which unfortified grain constituents are returned [16];
- folic acid degradation during production, baking, and storage [17];
- water loss from the dough during baking, which is higher in flat breads than in loaf breads;
- presence of naturally occurring free folic acid (if any).

### 3.2. Estimated Folic Acid Intakes—Target Group, Children, and Adults

Mean daily folic acid intake of the target population and of other sub-groups of the population increased after the introduction of mandatory folic acid fortification of wheat flour for making bread. The postfortification dietary folic acid intake of the target group was estimated to have increased by 159 *μ*g/day. The increase in intake was around 20% higher among the nontarget adult population because of the higher consumption of bread by men than women. For both adult groups, intake after fortification was around 2.5 times intake before fortification.

**Figure 1:**
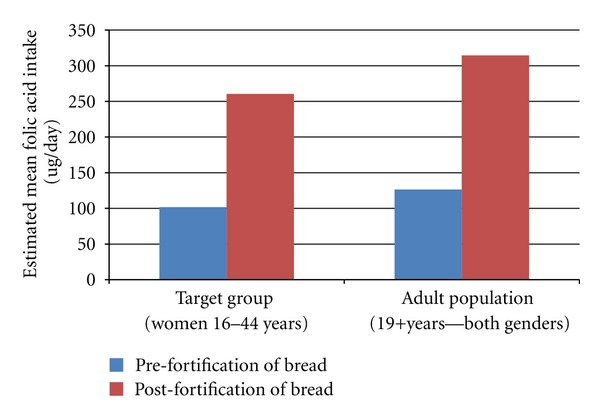
Estimated mean folic acid intake—target group and all adults.

The estimated increases in mean folic acid intake for the target group and other age groups of the Australian population, following mandatory folic acid fortification of wheat flour, are shown in Table 2 and Figure 1.

### 3.3. Estimated Tenth Percentile and Ninetieth (90th) Percentile Dietary Folic Acid Intakes

The tenth and ninetieth (90th) percentiles were selected to represent a usual low daily intake and a usual high daily intake of folic acid, respectively. The tenth and ninetieth (90th) percentile folic acid intakes for the target group and other population sub-groups, before and after mandatory folic acid fortification of bread, are given in Table 3. 

### 3.4. Comparison of Estimated Intakes with the Upper Level

The estimated dietary folic acid intakes were compared to the upper level of intake (UL–the highest average daily nutrient intake level likely to pose no adverse health effects to almost all individuals in the general population.) for folic acid for the target group and other age groups.  Table 4 shows the proportion of the target and the nontarget population groups with daily folic acid intakes above the UL for their age group. Only 1% or less of the target group and of all adults exceeded the folic acid UL before and after fortification. A higher proportion of children in the 2 to 16 years age group exceeded the UL after fortification than before fortification. The groups most affected were those aged 2–8 years.

## 4. Discussion

### 4.1. Amount of Folic Acid in Bread

The presence of measurable levels of folic acid in all the breads that are required to be made with fortified wheat flour indicates that this requirement has been implemented by bread manufacturers. The low levels of added folic acid in some gluten-free and organic breads indicate that there is some voluntary use of folic acid in these breads, which are not required to be made with folic-acid-fortified flour. 

The short sample collection period may generate some inaccuracies when used to estimate folic acid intakes, because the samples may not reflect longer-term fortification levels. There is also measurement uncertainty associated with folic acid analysis in this bread survey and in earlier surveys of voluntarily fortified foods.

The levels of folic acid measured in the breads surveyed were higher than what was predicted before the implementation of the standard. There may be several reasons for this, including flour fortification occurring at the upper end of the permitted fortification range rather than the midpoint, and the loss of folic acid on baking and storage being less than predicted. Additional sampling and analysis of breads at subsequent time points would be helpful for future monitoring. The same finding was observed in the US after the introduction of mandatory folic acid fortification in that country [18].

### 4.2. Folic Acid Intake Estimates

Using food consumption data from the 1995 NNS to estimate the folic acid intake in the target group introduces some additional uncertainty into the analysis, because consumption patterns may have changed since 1995. The true impact of mandatory fortification may therefore be different to what has been estimated here. The notably higher intakes seen in teenagers, estimated using 2007 consumption data, compared to adults estimated using 1995 data, suggest that current adult folic acid intakes may actually be higher than the estimated postfortification intakes. The differences may be due to an increased number of voluntarily fortified foods or to changes in the amounts of bread consumed between 1995 and 2007. The assumptions about market share and product formulation used to assign folic acid levels to voluntarily fortified foods and to mixed dishes in some of the data preparation could also introduce other inaccuracies.

To address some of these limitations estimates based on current Australian population food consumption data need to be undertaken in the future. The nutrition and physical activity component of the 2011–13 Australian Health Survey being conducted by the Australian Bureau of Statistics, will provide new and more appropriate population data on the amounts of food consumed and proportion of the population consuming breads and other fortified foods for future estimates. However, inaccuracies due to the use of a 24-hour recall method, such as over or under estimation of food consumption amounts, potentially affect all intake estimates provided [19]. 

### 4.3. Comparison of Estimated Folic Acid Intakes against Health Recommendations

Mandatory folic acid fortification of bread has resulted in women of child-bearing age having higher estimated intakes of folic acid and bringing their daily intake closer to 400 *μ*g/day, the amount recommended for a reduced incidence of NTDs. The estimated mean total daily intake was more than one-half of the recommended level when intakes of folic acid from mandatory and voluntarily fortified foods were taken into account. Among women whose usual intakes were low (represented by the tenth percentile of intake), mandatory fortification has had a particularly marked effect, increasing their estimated intake from less than 5% of that recommended to a quarter of the recommended amount. However, for the majority of the target group, dietary supplements would be required to achieve the recommended daily intake of folic acid.

The proportion of children aged 2–8 years that potentially exceed the UL for folic acid after mandatory fortification, was predicted in the pre-fortification estimates [1]. This prediction was a constraint on the level of folic acid that was mandated to be added to wheaten bread flour. Although the proportion of children who potentially exceed the relevant UL steadily decreases with age, the possible health risks of high folic acid intake require scrutiny particularly because the requirements for children are based on extrapolations from studies conducted with adults [20]. 

FSANZ is keeping a watching brief on research in this area, including the AIHW reports of all monitoring activities and the review process for the National Health and Medical Research Council Nutrient Reference Values for Australia and New Zealand, which the Australian Commonwealth Department of Health and Ageing has commenced. In addition, FSANZ will reassess folic acid and iodine intake levels for the Australian population (target and nontarget groups) when more up-to-date food consumption data from the 2011–2013 Australian Health Survey become available in late 2013.

## 5. Conclusions

Based on the recent survey of breads in the food supply, mandatory folic acid fortification of wheaten bread flour in Australia has increased the estimated dietary folic acid intake in women of child-bearing age (the target population). It has also brought about an increase in the estimated dietary intake of folic acid for nontarget population groups, irrespective of age.

The estimated increases in mean dietary folic acid intakes after fortification exceed the estimates made during development of the mandatory folic acid fortification standard. As predicted, the estimated increase in intakes in women of child-bearing age falls short of the recommended folic acid intake of 400 *μ*g folic acid per day, without additional use of dietary supplements. 

## Figures and Tables

**Table 1:** Measured amounts of folic acid in the different bread types.

Bread category	Number of samples	Median amount of folic acid (*μ*g/100 g)	Mean amount of folic acid (*μ*g/100 g)	Standard deviation (*μ*g/100 g)	Number of samples with no folic acid
White sandwich loaf	28	205	200	52	0
Flat breads/wraps	8	190	197	74	0
Wholemeal sandwich loaf	16	190	189	37	0
English muffin	8	165	180	33	0
Multigrain & seeds sandwich loaf	17	160	164	32	0
Organic	7	36	39	40	1
Gluten free	16	7	21	27	6

**Table 2:** Estimated folic acid intakes from foods and beverages before and after mandatory bread flour fortification.

Age group (years) both genders	Mean intake of folic acid (*μ*g/day)^#^		
	Pre-fortification	Post-fortification	Increase (and percentage increase) in mean dietary folic acid intake
2-3^¥^	104	227	123 (118%)
4–8^¥^	108	276	168 (156%)
9–13^¥^	107	307	200 (187%)
14–16^¥^	110	343	233 (212%)
**16–44 (women)** ^ ∗^	**102**	**261**	**159 (156%)**
19 & over^∗^	127	314	187 (147%)

^#^Does not include use of dietary supplements.

^¥^2007 ANCNPAS data: number of respondents aged 2-3 years = 552; 4–8 years = 1,520; 9–13 years = 1,493; 14–16 years = 922.

^∗^1995 NNS restricted sample: number of respondents aged 16–44 years (females only) = 328; 19 years and above (both genders) = 1,163.

**Table 3:** Estimated 10th and 90th percentile folic acid intakes from fortified foods  before and after the introduction of mandatory bread flour fortification.

	Folic acid intake (*μ*g/day)^#^			
Age group (both genders)	10th percentile		90th percentile	
	Pre-fortification	Post-fortification	Pre-fortification	Post-fortification
2-3 years^¥^	1.3	73	231	405
4–8 years^¥^	1.6	114	261	484
9–13 years^¥^	0.7	114	246	549
14–16 years^¥^	0	111	294	620
**16**–**44 years (women)** ^∗^	**11**	**79**	**251**	**475**
19 years & above^∗^	14	105	302	582

^#^Does not include use of dietary supplements.

^¥^2007 ANCNPAS data: number of respondents aged 2-3 years = 552; 4–8 years = 1,520; 9–13 years = 1,493; 14–16 years = 922.

^∗^1995 NNS restricted sample: number of respondents aged 16–44 years (females only) = 328; 19 years and above (both genders) = 1,163.

**Table 4:** Proportion of population (target group and non-target groups) with estimated folic acid intakes above the upper level.

Age group (both genders)	UL (*μ*g/day)	Proportion of respondents with folic acid intakes above the UL (%)^#^	
		Pre-fortification	Post-fortification
2-3 years^¥^	300	5	24
4–8 years^¥^	400	3	17
9–13 years^¥^	600	1	7
14–16 years^¥^	800	<1	5
19 years and above^∗^	1,000	<1	1
**16**–**44 years ** **(women)** ^∗^	Based on the relevant UL for each individual in the estimates—800 or 1000 depending on age	**0**	**<1**

^#^Does not include use of dietary supplements.

^¥^2007 ANCNPAS data: number of respondents aged 2-3 years = 552; 4–8 years = 1,520; 9–13 years = 1,493; 14–16 years = 922.

^∗^1995 NNS restricted sample: number of respondents aged 16–44 years (females only) = 328; 19 years and above (both genders) = 1,163.
